# Characterization of Fused Mn and Cu Corroles and Their Potency as ORR Electrocatalysts

**DOI:** 10.1021/acs.inorgchem.5c05722

**Published:** 2026-03-03

**Authors:** Sachin Kumar, Amit Kumar, Sruti Mondal, Arik Raslin, Anubhi Rawat, Natalia Fridman, Atif Mahammed, Zeev Gross

**Affiliations:** Schulich Faculty of Chemistry, 26747 Technion - Israel Institute of Technology, Haifa 320003, Israel

## Abstract

Given the rather poor activity of regular copper and manganese corroles as electrocatalysts for the oxygen reduction reaction (ORR), we now report the synthesis, structural characterization, and catalytic activity of binuclear manganese and copper complexes coordinated to β–β dimeric corrole frameworks. X-ray crystallographic analysis reveals distinct coordination environments and structural flexibility in these bimetallic systems: an almost perfect square geometry for the 4-coordinate (no axial ligands) bis-copper complex, while 5- and 6-coordinate structures were fully characterized for manganese with varying degrees of corrole planarity and metal displacement. The conjugation between the corrole subunits differs quite meaningfully for the copper and manganese complexes, which comes into play by much shorter near-IR bands in the electronic spectrum of the former, significantly longer C–C bond lengths in the bridging moiety of the latter, and differences in their redox potentials relative to the respective mononuclear complexes. Examination of porous carbon electrodes modified by these molecular ORR catalysts uncovered the superior performance of the binuclear manganese corrole dimer.

## Graphical abstract

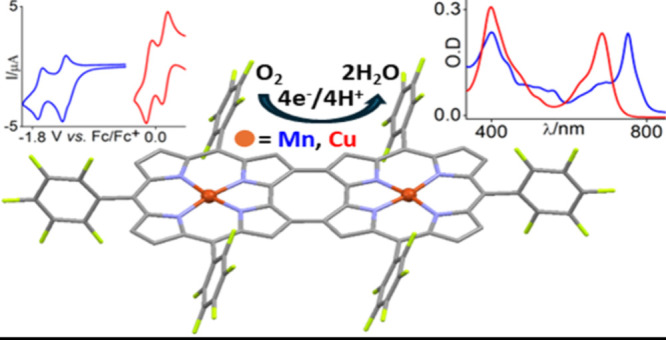

## Introduction

Macrocyclic π-conjugated systems have attracted significant research interest as building blocks for advanced materials with electronic and optical applications. Beyond their materials science applications, these aromatic frameworks demonstrate exceptional versatility in coordinating metal centers and forming sophisticated supramolecular architectures.^1^ The concept of bimetallic catalysis has gained prominence in modern synthetic chemistry, where dual metal centers can function through either cooperative or sequential mechanisms. This approach frequently delivers enhanced catalytic performance through improved turnover rates, superior selectivity profiles, and access to previously inaccessible reaction pathways.^2^ Nature provides compelling examples of this strategy, as evident by the prevalence of multimetallic active sites in crucial enzymes, including tyrosinase, superoxide dismutase, methane monooxygenase, ribonucleotide reductase, urease, and various phosphohydrolases.^3^ Contemporary research is increasingly focused on metallocorrole coordination complexes featuring earth-abundant transition metals as viable catalysts for critical energy conversion processes. These systems have demonstrated promise in oxygen reduction reactions, water splitting processes, and carbon dioxide valorization.^4^ The maturation of synthetic protocols for corrole ligand preparation has significantly expanded their utility across diverse fields, including heterogeneous catalysis, chemical sensing, and photonic materials.^5^ The transition toward sustainable energy systems has positioned fuel cell technology as a critical alternative to traditional combustion-based power generation, particularly for mobile and transportation applications.^6^ However, the widespread adoption of proton exchange membrane fuel cells (PEMFCs) still faces substantial technical challenges, most notably the intrinsically slow kinetics governing the cathodic oxygen reduction reaction (ORR).^7^ A fundamental requirement for effective ORR catalysis is achieving high selectivity for the ideal four-electron reduction pathway (O_2_ + 4H^+^ + 4e^–^ → 2H_2_O) while minimizing the competing two-electron pathway that generates hydrogen peroxide (O_2_ + 2H^+^ + 2e^–^ → H_2_O_2_). The formation of hydrogen peroxide is particularly problematic as it leads to progressive degradation of both catalytic materials and fuel cell infrastructure.^8^ Currently, platinum group metals represent the state-of-the-art for ORR electrocatalysis, offering exceptional 4-electron selectivity, minimal overpotentials, and robust long-term performance.^9^ Nevertheless, their limited availability and prohibitive costs have driven intensive research toward economically viable alternatives. Within this context, first-row transition-metal complexes of N_4_ macrocycles like porphyrins, phthalocyanines, and corroles have emerged as particularly promising candidates.^10–12^ The performance of these molecular catalysts can be significantly enhanced through strategic immobilization on high-surface-area carbon supports, such as carbon nanotubes or porous carbon materials, which provide optimal conditions for electron transfer and reactant accessibility.^13–17^ A comparative study of first-row transition-metal corrole complexes for electrocatalytic oxygen reduction demonstrated a trend in activity, increasing in the order: cobalt > iron ≫ nickel > manganese > copper. Limited studies have explored thermally treated cobalt corrole systems supported on porous carbon electrodes, demonstrating encouraging power densities in polymer electrolyte fuel cell configurations.^18^ However, even though pyrolysis can be applied to macrocyclic MN_4_ complexes, it requires excessively high temperatures and inevitably compromises the preservation of the original structure.^19^

Regarding corroles and ORR catalysis, the focus was directed toward carbon electrodes modified by the corresponding cobalt complexes,^4a,20^ given the above-mentioned relatively poor ORR performance of mononuclear manganese and copper corroles. The goal of the current investigation was to explore the binuclear complexes of corrole dimers to assess any possible improvement in catalytic potency. Leveraging recent synthetic developments in β–β-linked corrole dimer construction,^21–23^ we present here the bis-manganese and bis-copper complexes of a dimeric corrole framework, which features at its center a cyclooctatetraene (COT) architecture (Scheme 1). They were fully characterized and analyzed by X-ray crystallography accompanied by focusing on differences in their electronic spectra and redox potentials relative to each other and their respective mononuclear corroles (Figures 1–3 and Figures S1–S9). Investigation of oxygen reduction catalysis was performed in alkaline aqueous solutions with a porous carbon cathode material modified by the complexes. This uncovered that those containing the binuclear manganese dimer exhibit enhanced activity, lower onset potentials, and reduced hydrogen peroxide formation.

**Scheme 1:**
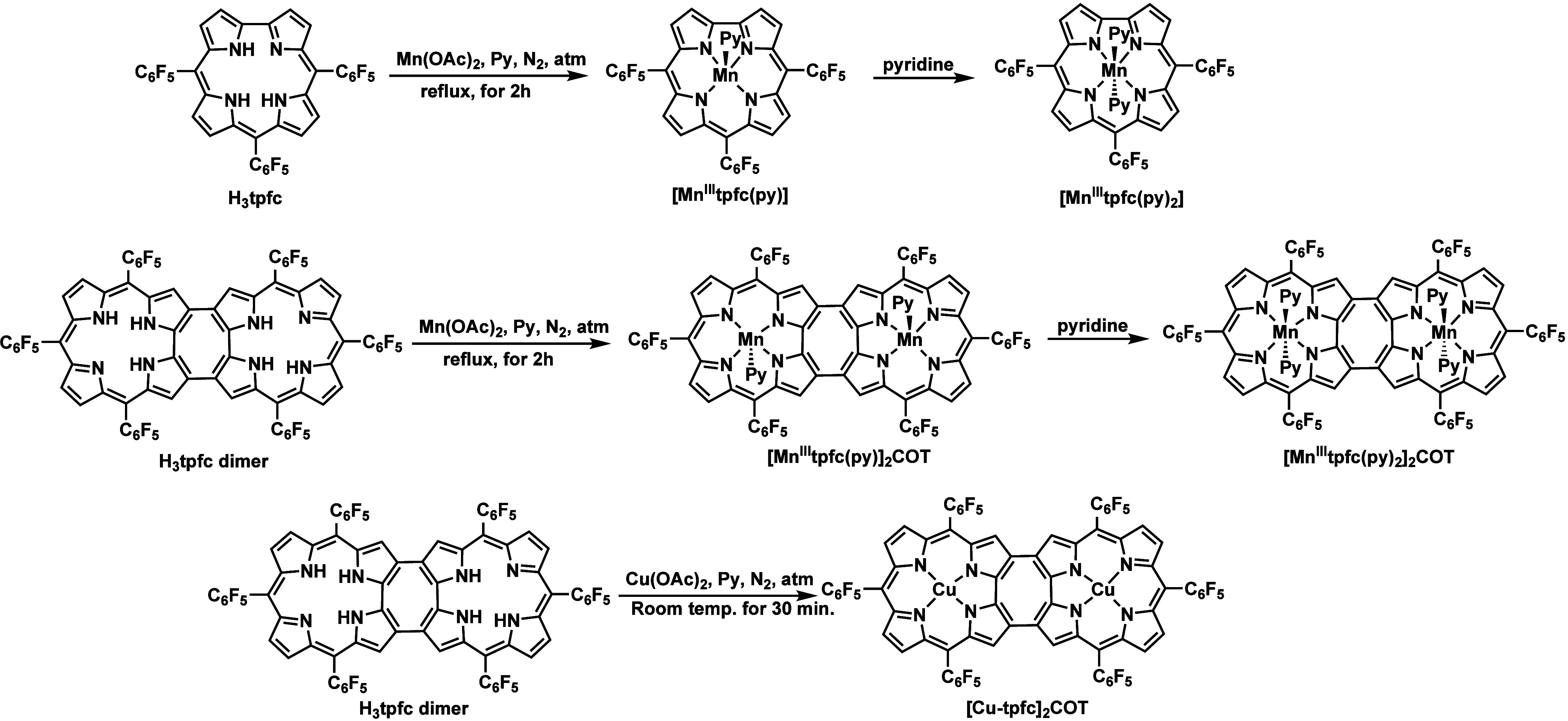
Synthesis of the Mononuclear and Binuclear Manganese and Copper Corrole Complexes

**Figure 1:**
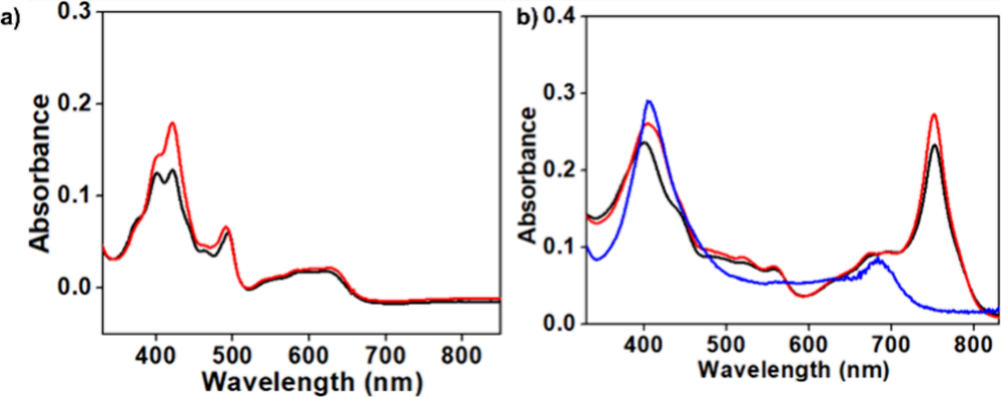
UV–vis spectra (in DCM, at 295 K) of (a) the mononuclear manganese complexes [Mn^III^tpfc­(py)] and [Mn^III^tpfc­(py)_2_] (black and red traces, respectively) and (b) the binuclear copper complex [Cu-tpfc]_2_COT (blue trace) and [Mn^III^tpfc­(py)]_2_COT (black trace) and [Mn^III^tpfc­(py_2_)]_2_COT (red trace) with 1 and 2 pyridine axial ligands for each manganese center, respectively.

## Results and Discussion

### Synthesis and Spectroscopic Characterization

The synthesis of manganese and copper corrole complexes was accomplished by relying on previous reports regarding the free-base H_3_tpfc and the corresponding H_3_tpfc dimer.^5j,22^ Standard metalation procedures involved treating pyridine solutions of the free-base corroles with Mn­(OAc)_2_ or Cu­(OAc)_2_ for 2 h under a reflux condition and room temperature, respectively.^24,25^ Following solvent removal and chromatographic purification, the target complexes were isolated in their pure form (Scheme 1). The product isolated upon manganese insertion into the H_3_tpfc dimer was [Mn^III^tpfc­(py)]_2_COT, with one pyridine axial ligand coordinated to each metal ion, which was transformed to [Mn^III^tpfc­(py)_2_]_2_COT when dissolved in pure pyridine. The new metal complexes were characterized by UV–visible and single-crystal XRD (Figures 1 and 2, Table 1, and Table S1, see the Supporting Information). The UV–vis spectra of the mononuclear manganese­(III) corrole complexes align well with previously reported data: typical split Soret bands (λ = 400–421 nm), a ligand-sensitive absorption around 490–493 nm, and broad Q bands near 600 nm (Figure 1a and Figure S1). Differences between the 5- and 6-coordinated complexes are minor, with only the somewhat more intense Soret band in the latter complex (Figure 1a, the black and red traces, respectively). The spectra of the binuclear Mn­(III) corrole complexes are very different: a nonsplit Soret band at about 400 nm, nondistinctive charge-transfer bands between 480 and 590 nm, and most pronounced intense near-infrared (NIR) absorptions with a maximum at λ = 751 nm (Figure S2 and Figure 1b, black and red traces for the complexes with 1 and 2 pyridine axial ligands for each manganese center, respectively). The latter feature is reminiscent of observations for both the free-base dimer and its Ga­(III) complexes: these display a low-energy band (λ_max_ at 720–724 nm) that were analyzed as an indication of extensive π delocalization through the COT bridge and the corresponding contribution to unique HOMO–LUMO transitions.^21^ The UV–vis spectrum of binuclear copper complex [Cu^III^tpfc]_2_COT (Figure 1b, blue trace) is characterized by a single Soret band similar to those of the manganese complexes but without charge-transfer bands and a NIR band that is at much higher energy (λ_max_ = 684 nm).

**Figure 2:**
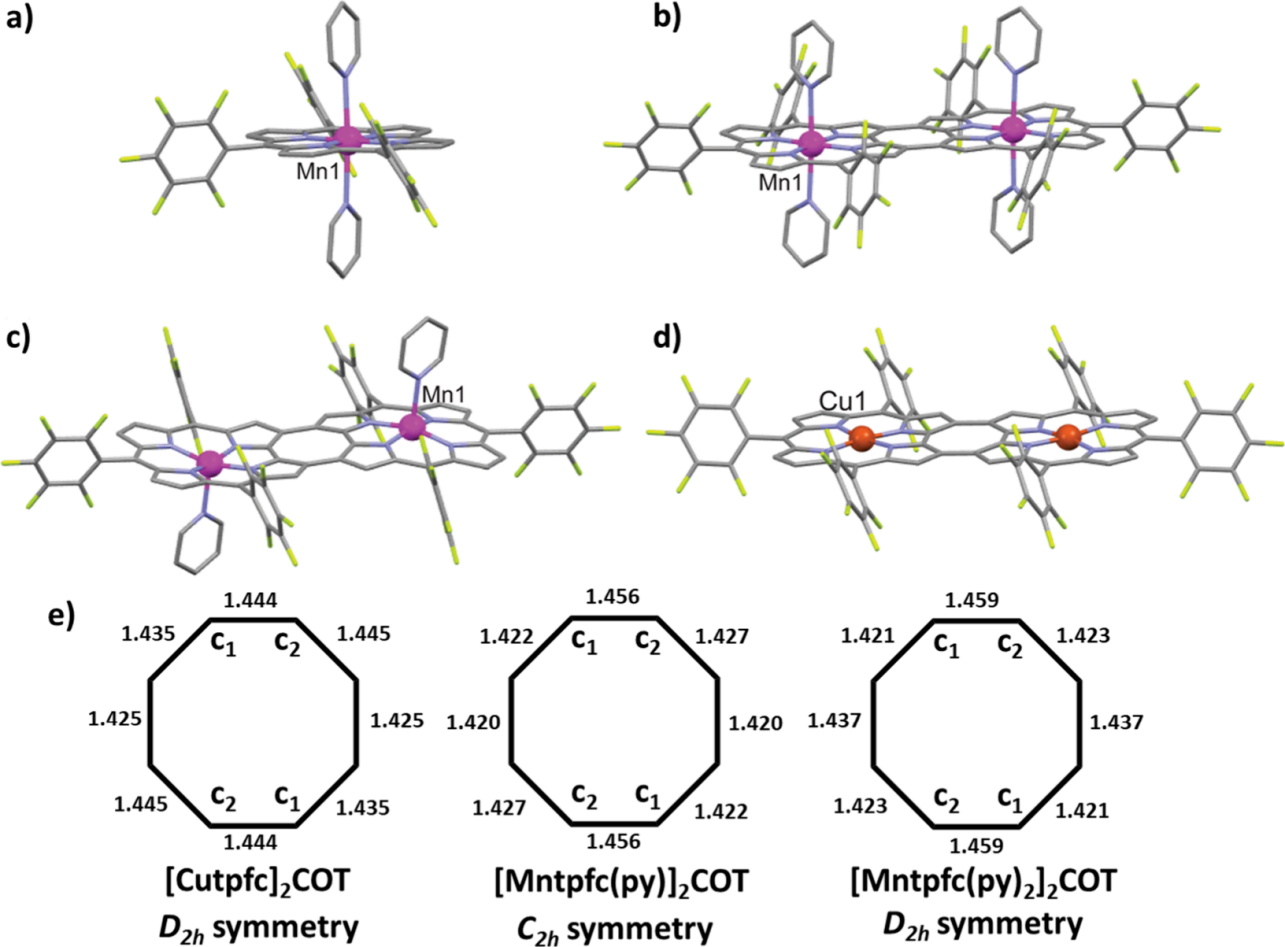
Side-view X-ray crystal structures of (a) [Mn^III^tpfc­(py)_2_], (b) [Mn^III^tpfc­(py)_2_]_2_COT, (c) [Mn^III^tpfc­(py)]_2_COT, and (d) [Cu-tpfc]_2_COT, in which all hydrogen atoms have been omitted for clarity, and (e) the C–C bond lengths within the COT moiety of the binuclear manganese and copper corrole complexes, with the C atoms that are bridging (and are not part of) the corrole subunits labeled C_1_ and C_2_ in accord with the symmetry groups. Note: Disorder is observed only in [Cu-tpfc]_2_COT. The C_6_F_5_ groups at the *meso* C10 and C10′ positions are disordered with occupancies of 0.51:0.49, and the *n*-hexane solvent molecule is disordered with occupancies of 0.72:0.28.

**Table 1:** Selected Structural Parameters of M­(III) Corroles

		**M–L**_**axial**_ ^a^		**M–N**_**avg**_ ^b^	**Δ*M***_**4**_ ^c^ **(Å)**	**Δ*M***_**23**_ ^c^ **(Å)**
**complex**		**L**_**1**_	**L**_**2**_	**M–N**_**avg**_ ^b^	**Δ*M***_**4**_ ^c^ **(Å)**	**Δ*M***_**23**_ ^c^ **(Å)**
[Mn^III^tpfc(py)_2_]		2.435(2)	2.418(2)	1.913(8)	0.003	0.003
[Mn^III^tpfc(py)]_2_COT	Mol-I	2.219(3)		1.915(9)	0.254	0.394
	Mol-II	2.219(3)		1.915(9)		
[Mn^III^tpfc(py)_2_]_2_COT	Mol-I	2.438(3)	2.428(3)	1.908(10)	0.000	0.004
	Mol-II	2.438(3)	2.428(3)	1.908(10)		
[Cu-tpfc][Bibr ref29]				1.889(8)	0.003	0.021
[Cu-tpfc]_2_COT	Mol-I			1.881(10)	0.004	0.009
	Mol-II			1.881(10)	0.004	0.009

a M–L_axial_: M–N­(pyridine) bond lengths for the 6- and 5-coordinate Mn­(III) corroles.

b M–N_avg_: average M–N bond length with the four equatorial corrole N atoms.

c Δ*M*_4_ and Δ*M*_23_: deviation of the metal ion from the mean plane defined by the 4-core nitrogen atoms and the 23-core atoms, respectively.

### Structural Analysis of the Binuclear Copper and Manganese Complexes of the Corrole Dimer

#### 4-Coordinate Cu Complex: [Cu-tpfc]_2_COT

The binuclear copper complex serves as an important structural reference within the series, providing insight into the intrinsic geometric features of the corrole framework in the absence of axial ligation. It adopts the typical for d^8^ square-planar coordination geometry, and its group symmetry is *D*_2h_. (Figure 2) Each copper center remains essentially coplanar with the macrocycle, exhibiting only a minimal out-of-plane displacement of 0.009 Å, which preserves the planarity of the corrole core commonly observed in 4-coordinate metallocorroles. The average Cu–Nc bond length of 1.881(10) Å is nearly identical to that reported for monomeric copper corroles (1.889(8) Å), confirming that dimerization does not significantly alter the local metal–ligand bonding environment. In terms of electronic configuration, we note that the mononuclear Cu^III^(tpfc) complex is ^1^H and ^19^F NMR-active and, importantly, observable ^1^H NMR and ^19^F spectra are also obtained for the binuclear [Cu-tpfc]_2_COT complex (Figures S5–S8). Temperature-sensitive variations in the chemical shifts and resonance broadning of various mononuclear copper corroles were one of the many indications that led to the current consensus: they are neither pure Cu­(III) (expected to be diamagnetic) nor (corrole-radical)­Cu­(II) (in singlet or triplet states) complexes, but rather exhibit a multiconfigurational electronic structure that is intermediate between these extremes.^26–28^ Within this framework, the NMR observability of the [Cu-tpfc]_2_COT dimer can be rationalized by effective electronic coupling between the two copper–corrole subunits through the cyclooctatetraene bridge.

#### 5-Coordinate Mn Complex: [Mn­(III)­tpfc­(py)]_2_COT

The 5-coordinate manganese dimer exhibits distinctive structural features: each manganese center adopts a 5-coordinate square-pyramidal geometry with a significantly domed corrole macrocycle conformation, and the axial pyridine ligands are anti to each other. The latter aspect is consistent with steric considerations and previously reported structures of analogous bis-metallic corrole systems.^21,23^ Analysis of the coordination environment reveals Mn–N_py_ bonds [2.219(3) Å] that are about 0.2 Å shorter than in 6-coordinate *trans*-pyridine complexes (*vide infra*) as may be expected from the absence of a trans influence and stronger π-back bonding interactions in the former case.^24,30,31^ The substantial metal displacement of 0.394 Å out of the C_19_N_4_ mean plane toward the axial pyridine is also consistent with the strong interaction between them. In contrast, the Mn–N_c_ distances [1.915(9) Å] are marginally elongated compared with monomeric systems.

#### 6-Coordinate Mn Complex: [Mn^III^tpfc­(py)_2_]_2_COT

This complex has structural features that highlight the coordination flexibility of these systems. Each manganese center adopts an octahedral-like geometry with two axial pyridine ligands in which the metal is displaced by only 0.004 Å from the nearly planar corrole macrocycle conformation. The two axial pyridines exhibit distinct bond lengths to the manganese center, 2.428(3) and 2.438(3) Å, very similar to those observed in the monomeric analogue (Mn^III^tpfc)­(py)_2_ [2.418(2) and 2.435(2) Å], suggesting that the first coordination sphere remains locally unperturbed and behaves analogously to discrete mononuclear units. Relative to the 5-coordinate [Mn^III^tpfc­(py)]_2_COT, the axial Mn–N_py_ bonds are about 0.2 Å longer (*vide supra*) while the equatorial bonding Mn–N_c_ distances [1.908(10) Å] are shorter by about 0.07 Å. Overall, these comparisons demonstrate how ligand-induced geometric modulation governs metallocorrole architecture.^32–34^

A detailed structural examination of the cyclooctatetraene (COT) bridging unit could potentially provide valuable insight into the interactions between the two corrole subunits. Common to both the 5- and 6-coordinate manganese dimers is that the bonds that are not part of the corroles (C_1_–C_2_ in Figure 2e) are significantly longer (145.6 and 145.9 pm, respectively) than all other ones (142.0–142.7 and 142.1–143.7 pm, respectively). This is not the case for the 4-coordinate copper complex, in which all C–C bond lengths are much more similar to each other, in a rather small range of 144.5–142.5 pm. This may suggest that π-conjugation between the two corrole subunits is most effective in the latter case in terms of being fully delocalized over the entire dimeric framework.

### Electrochemical Properties of the Mono- and Binuclear Cu and Mn Corroles

The cyclic voltammograms (CVs) (Figure 3) of the mononuclear [Mn^III^tpfc­(py)_2_] and binuclear [Mn^III^tpfc­(py)_2_]_2_COT were recorded in a pyridine solvent (rather than more commonly used CH_3_CN) to ensure that they remain 6-coordinate in solution (Figure 3a). The focus was on the reduction processes occurring between −0.8 and −1.9 V vs Fc/Fc^+^, to elucidate the effect of the bridge on the reduction potential. Of note is that all processes were reversible, which indicates that the pyridine axial ligands remain coordinated after reduction. The CV of [Mn^III^tpfc­(py)_2_]_2_COT displays two well-separated redox couples with half-wave potential (*E*_1/2_) values of −1.34 and −1.69 V (Δ = 0.35 V), while the *E*_1/2_ for the Mn^III^/Mn^II^ process for [Mn^III^tpfc­(py)_2_] is in between, −1.54 V. This implies that the first of the two reductions is easier for the dimer because of the larger π-system therein, which would be relevant even if the process is metal-centered and not corrole-centered, while the second reduction is harder because it takes place in an already charged system.^21,23^ The results obtained for the copper complexes were very different (Figure 3b): the two redox potentials for [Cu-tpfc]_2_COT are still well separated (Δ = 0.167 V), but both are obtained at less negative potentials (*E*_1/2_ = −0.034 and −0.17 V) than for the mononuclear [Cu-tpfc]^−^/[Cu-tpfc] redox process (*E*_1/2_ = −0.18).

**Figure 3:**
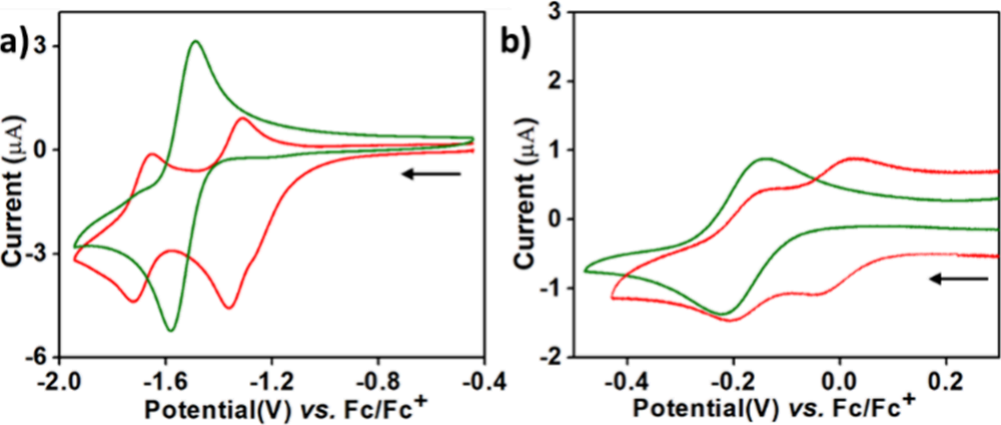
Cyclic voltammograms (0.5 mM) of (a) mononuclear [Mn^III^tpfc­(py)_2_] (green) and binuclear [Mn^III^tpfc­(py)_2_]_2_COT (red) in pyridine as a solvent and (b) mononuclear [Cu-tpfc] (green) and binuclear [Cu-tpfc]_2_COT (red) in CH_3_CN recorded under a N_2_ atmosphere, with tetrabutylammonium perchlorate (TBAP, 0.1 M) as a supporting electrolyte, at a scan rate of 100 mVs^–1^. Potentials are listed versus the Fc/Fc^+^ couple determined under identical conditions.

A plausible explanation for this phenomenon relies on two differences: (a) copper corroles are known to be the most prominent case of the noninnocent ligand, i.e., a very strong contribution of copper­(II) coordinated by a corrole radical relative to the alternative copper­(III)-nonoxidized corrole structure;^28,35^ (b) the above-mentioned indications about outstandingly large delocalization between the corrole subunits through the COT bridging part. The existence of an electronic structure with significant diradical contribution could explain why even reduction by two electrons occurs at a very low potential. An alternative explanation relies on the almost identical C–C bond lengths of 1.445–1.425 Å (Δ = 20 pm rather than 36 and 38 pm in the other complexes) without any obvious bond alternation in the bridging unit present in [Cu-tpfc]_2_COT. This suggests that its macrocyclic system is completely different, which is consistent with noting that the electron count of π electrons in the dimer involving delocalization of the COT moiety is antiaromatic and that addition or removal of two electrons would render it aromatic.^1a^

#### Heterogeneous ORR in Basic Water

Building on the understanding gained from prior investigations, we evaluated the performance of the synthesized complexes as heterogeneous electrocatalysts for the oxygen reduction reaction (ORR) in aqueous media (Figure 4). The complexes were immobilized onto a high-surface-area carbon support, Black Pearls 2000 (BP2000),^36^ following established procedures (Figures S9 and S10).^13^ Specifically, 10 mg of BP2000 was dispersed in 1 mL of isopropyl alcohol solutions of the complexes (0.8 mg/mL) and stirred to promote adsorption (see the Supporting Information for the detailed methodology). Interestingly, similar treatment with free-base corroles also resulted in efficient binding, suggesting that the primary driving force behind adsorption is π–π stacking interactions between the corrole π-system and the graphitic surface of BP2000.^20^ Of note is that immobilization of [Mn^III^tpfc­(py)_2_]_2_COT was significantly more efficient than that of the corresponding monomer [Mn^III^tpfc­(py)_2_] (100% vs 75%, respectively, Figure S9).

**Figure 4:**
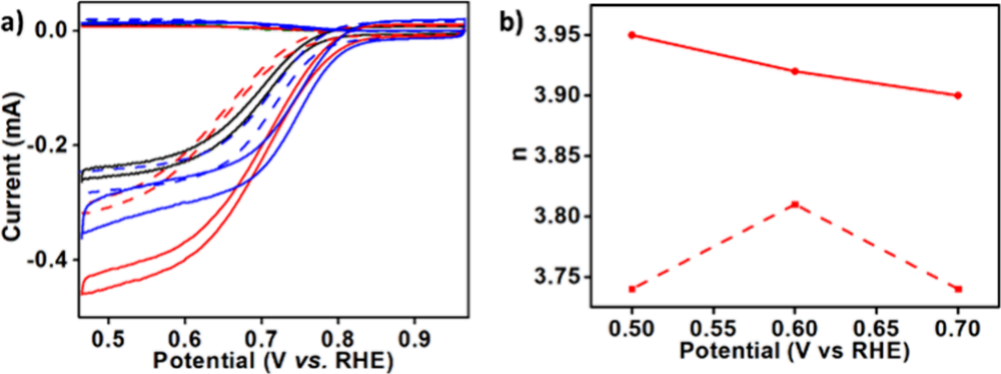
(a) RRDE traces obtained by performing ORR catalysis under alkaline conditions (0.1 M KOH) by using BP2000 electrodes that are either nonmodified (black) or modified by [Mn^III^(tpfc)­(py)_2_] (red dotted), [Mn^III^(tpfc)­(py)_2_]_2_COT (red solid), [Cu­(tpfc)] (blue dotted), and [Cu­(tpfc)]_2_COT (blue solid); (b) the number of transferred electrons during ORR catalysis performed by the electrodes modified by [Mn^III^(tpfc)­(py)_2_] (dotted) and [Mn^III^(tpfc)­(py)_2_]_2_COT (solid).

The ORR activity of the catalyst-modified carbon materials was assessed under alkaline conditions (0.1 M KOH), focusing on two key performance indicators: onset potential and product selectivity (water vs hydrogen peroxide), as shown in Figure 4. Previous investigations of metallocorroles for ORR electrocatalysis uncovered that the copper and manganese complexes displayed the lowest activity.^4a,37^ The results presented in Figure 4a reveal that the improvements regarding onset potential and catalytic current by changing from mono- to binuclear corroles (broken and full lines, respectively) are quite small for copper (blue) but very significant for manganese (red). A comparison between the mono- and binuclear manganese complexes uncovered a much larger selectivity of the latter for the desirable 4e^–^/4H^+^ route, producing less than 3% H_2_O_2_. This is presented in Figure 4b in terms of the number of transferred electrons being much closer to 4 for catalysis by [Mn^III^(tpfc)­(py)_2_]_2_COT rather than by [Mn^III^(tpfc)­(py)_2_].

## Conclusions

Three metal bis-corrole complexes were synthesized and thoroughly characterized: 4-coordinate [Cu­(tpfc)]_2_COT, 5-coordinate [Mn^III^(tpfc)­(py)]_2_COT, and 6-coordinate [Mn^III^(tpfc)­(py)_2_]_2_COT, containing zero, one, and two axial pyridine ligands per metal center, respectively. X-ray crystallographic analyses revealed a dome-shaped geometry for the monopyridine Mn complex, while both the Mn bis-pyridine and the pyridine-free Cu complexes exhibited nearly planar structures. Electrochemical studies revealed that the 1-electron reduction of the binuclear complexes is more facile (i.e., requires less negative potential) than that of their corresponding mononuclear complexes, and that even the 2-electron reduction of the binuclear Cu complex is more facile than the reduction of the mononuclear corrole by one electron. Analyses of the corrole-bridging COT moiety also point to significant differences in the participation of π-electron delocalization in the case of the Cu dimer, which further comes into play by a much less red-shifted near-IR band. Utilization of the new complexes as ORR electrocatalysts uncovered almost no improvement of the bi- relative to mononuclear Cu corroles, but the binuclear Mn complexes do exhibit superior catalytic performance in terms of a more positive onset potential, larger catalytic currents, and lower H_2_O_2_ yield compared to the corresponding monomer. This likely reflects the combination of stronger absorbance and the cooperative effect of two nearby metal ions with proper reduction potentials.

## Experimental Section

### Chemicals and Instrumentation

All common chemical reagents and solvents were purchased from commercial sources and purified before use according to established protocols. Dichloromethane A.R., *n*-hexanes A.R., diethyl ether extra dry, and HPLC-grade acetonitrile were purchased from J.T. Baker. Pyrrole (99%, extra pure), 1,2,4-trichlorobenzene, Mn­(OAc)_2_.4H_2_O, Cu­(OAc)_2_.H_2_O (for metalation), tetrabutylammonium hexafluorophosphate (≥99%), and Nafion (5%) were purchased from Sigma. Pentafluorobenzaldehyde was purchased from Apollo Scientific. Pyridine and heptane (for analysis) were purchased from Mercury. Oxygen, and nitrogen gases with 99.999% purity were purchased from Maxima. BP2000 was purchased from the Fuel Cell Store company. The silica gel used for column chromatography was Kiesel gel 60, 230–400 mesh. Absorption spectra of synthesized corroles were recorded on an Agilent Technologies Cary 8454 UV–vis spectrophotometer. Quartz cuvettes of 1.0 cm thickness were used to measure the samples. High-resolution mass spectra for the compounds were acquired on a Bruker MaXis Impact mass spectrometer, using an APCI (atmospheric pressure chemical ionization) direct probe in either positive or negative mode. The cyclic voltammetry experiments were performed on a PALMSENS EmStat3+ potentiostat. The RRDE electrochemistry was performed on a Bio-Logic VSP bipotentiostat.

### Synthesis

The synthesis of free-base H_3_tpfc, COT bridge corrole dimer, and their metalation was carried out according to earlier reported procedure.^5j,21,25^

### Synthesis of 5,10,15-Tris­(pentafluorophenyl)­corrolato Manganese­(III) Pyridine, [Mn^III^tpfc­(py)_2_]

A pyridine solution (20 mL) of H_3_tpfc (20 mg, 0.025 mmol) and manganese­(II) acetate tetrahydrate (123 mg, 0.5 mmol) was heated to reflux for 2 h under nitrogen. The solvent was evaporated, and the residue was passed through a column of silica with hexane/dichloromethane/pyridine (75:20:5) as eluent, resulting in 19.92 mg (86% yield) of 2-Mn­(py). Dark-green X-ray-quality crystals were obtained by slow evaporation of a solution of [Mn^III^tpfc­(py)_2_] in benzene/*n*-heptane. UV–vis (DCM): λ_max_ (ε, M^–1^cm^–1^): 401­(11,600), 421­(11,800), 493(5500), 557(1000), 589(1700), 622(1700) nm. HRMS (APCI, positive mode) *m*/*z*: calculated for C_37_H_8_F_15_MnN_4_: 847.9889 [M–2py+H]^+^; observed 848.9987.

### Synthesis of [Mn^III^tpfc­(py)]_2_COT with One and Two Pyridine Axial Ligand on Each Manganese Center

The free-base corrole dimer (20 mg, 0.0125 mmol) was dissolved in 10 mL of pyridine under N_2_ followed by the addition of manganese­(II) acetate tetrahydrate (62 mg, 0.25 mmol) and heated to reflux for 2 h. The solvent was evaporated, and the residue was passed through a column of silica with hexane/dichloromethane/pyridine (75:20:5) as eluent, resulting in 16.17 mg (70% yield) of [Mn^III^tpfc­(py)]_2_COT. Dark-green-colored X-ray-quality crystals were obtained by slow evaporation of a solution of [Mn^III^tpfc­(py)]_2_COT in benzene/*n*-heptane. UV–vis (DCM): λ_max_ (ε, M^–1^cm^–1^): 399­(27,000), 520(9200), 557(9300), 693­(10,800), 751­(26,700) nm. HRMS (ESI, negative mode) *m*/*z*: calculated for C_74_H_12_N_8_F_30_Mn_2_.H_2_O: 1709.9571 [M–2py+H_2_O]^−^; observed 1710.0178. [Mn^III^tpfc­(py)_2_]_2_COT was obtained by dissolving [Mn^III^tpfc­(py)]_2_COT in pure pyridine and subsequent evaporation of the solvent.

### Synthesis of [Cu-tpfc]_2_COT

The free-base corrole dimer (10 mg, 0.0062 mmol) was dissolved in 10 mL of pyridine under N_2_, followed by the addition of copper­(II) acetate monohydrate (50 mg, 0.25 mmol), and stirred at room temperature for 30 min. The solvent was evaporated, and the residue was passed through a column of silica with hexane/dichloromethane (70:30) as eluent, resulting in 5.72 mg (54% yield) of [Cu-tpfc]_2_COT. Dark-green-colored X-ray-quality crystals were obtained by slow evaporation of a solution of [Cu-tpfc]_2_COT in DCM/hexane. UV–vis (DCM): λ_max_ (ε, M^–1^cm^–1^): 404 (11,000), 684(2200) nm. ^1^H NMR (377 MHz, CDCl_3_) δ 6.96 (s, 4H), 6.65 (d, *J* = 4.0 Hz, 4H), 6.55 (d, *J* = 4.0 Hz, 4H) ppm. ^19^F NMR (377 MHz, CDCl_3_) δ −136.20 (dd, *J* = 22.5, 6.1 Hz, 4F), −137.40 (d, *J* = 16.0 Hz, 2F), −151.28 (t, *J* = 21.0 Hz, 2F), −151.60 (t, *J* = 20.8 Hz, 1F), −159.94 to −160.14 (m, 6F). HRMS (ESI, negative mode) *m*/*z*: calculated for C_74_H_12_N_8_F_30_Cu_2_: 1707.9292 [M]^−^; observed 1707.9878.

### Adsorption of Corroles on BP2000 and Ink Preparation for Working-Electrode Modification

Metallocorrole (0.8 mg) was dissolved in 1 mL of IPA by 5 min of sonication. BP2000 (10 mg) was added, and the solution was sonicated for 15 min and left to stir for 24 h. After that, the catalyst was centrifuged for 30 min, the solution was separated, and the carbon support was dried at 45 °C in an oven overnight. Then, 1 mL of IPA was added to the solid material, and the mixture was centrifuged again. The solution was removed and combined with the previous day’s solution to test how much corrole did not adsorb to BP2000. The catalyst containing BP2000 was dried again at 45 °C in an oven overnight. The ink consisted of 1 mg of modified carbon, 0.2 mL of IPA, 0.8 mL of DI water, and 10 μL of Nafion. The mixture was sonicated for 30 min. The ink (5.0 μL) was drop-cast on the working electrode and dried under air for 30 min and then at 45 °C in the oven for 30 min.

## Supplementary Material



## Notes

UV–vis spectra, single-crystal X-ray data, HRMS, and ^1^H and ^19^F NMR spectra of the compounds (PDF)

## #

Institute of Inorganic Chemistry, University of Goettingen, Goettingen 37077, Germany (A.K.)
