# Supplementary material for: Characterization of Fused Mn and Cu Corroles and Their Potency as ORR Electrocatalysts
Source: Inorg Chem. 2026 Mar 3;65(10):5552–9. doi: 10.1021/acs.inorgchem.5c05722 (PMC12997157; doi:10.1021/acs.inorgchem.5c05722)
Supplement: Supplementary file 1 [file ic5c05722_si_001.pdf]

Supporting information

Characterization of Fused Mn and Cu Corroles and Their Potency as ORR Electrocatalysts

Sachin Kumar,[1] Amit Kumar,[1#] Sruti Mondal,[1] Arik Raslin,[1] Anubhi Rawat,[1] Natalia Fridman,[1] Atif Mahammed,[1] and Zeev Gross[1]*

[1]Schulich Faculty of Chemistry, Technion–Israel Institute of Technology, Haifa 320003, Israel. Email: chr10zg@technion.ac.il

[#] Current address: Institute of Inorganic Chemistry, University of Goettingen, Goettingen, Germany

Table of Contents	Page
Crystallographic details	S3
**Table S1:** Crystallographic information for all complexes.	S4
**Electrochemical Experiments**	S6
**Figure S1**: UV-vis (in CH_2Cl_2 at 295 K) spectra of free base H_3tpfc (Back line) and [Mn^{III}tpfc(py)$_2$] (red line).	S6
**Figure S2**: UV-vis (in CH_2Cl_2 at 295 K) spectra of free base dimer (blue line) and [Mn^{III}tpfc(py)]$_2$COT (red line).	S7
**Figure S3**: ^{1}H NMR of free-base (H_3tpfc COT) dimer in $CDCl_3$.	S7
**Figure S4**: ^{19}F NMR of free-base (H_3tpfc COT) dimer in $CDCl_3$.	S7
**Figure S5**: ^{1}H NMR of Cu(tpfc) monomer in $CDCl_3$.	S8
**Figure S6**: ^{19}F NMR of Cu(tpfc) monomer in $CDCl_3$.	S8
**Figure S7**: ^{1}H NMR of [Cu(tpfc)]$_2$COT dimer in $CDCl_3$.	S9
**Figure S8**: ^{19}F NMR of [Cu(tpfc)]$_2$COT dimer in $CDCl_3$.	S9
**Figure S9:** Electronic spectra before (blue traces) and after (red traces) adsorption of 0.8 mg of (a) [Mn^{III}tpfc(py)$_2$] and (b) [Mn^{III}tpfc(py)]$_2$COT on 10 mg of BP2000 from 1 mL solution of isopropanol.	S10
**Figure S10:** Electronic spectrum before (blue traces) and after (red traces) adsorption of 0.8 mg of [Cu-tpfc]$_2$COT on 10 mg of BP2000 from 1 mL solution of isopropanol.	S11
**Figure S11:** UV-vis (in CH_2Cl_2 at 295 K) spectra of free base H_3tpfc (blue line) and (H_3tpfc COT) dimer (red line).	S11
**Figure S12.** MS (APCI positive mode) of H_3tpfc.	S12
**Figure S13**: HRMS (APCI positive mode) of [Mn^{III}tpfc(py)$_2$].	S13
**Figure S14**: HRMS (APCI positive mode) of (H_3tpfc COT) dimer.	S14
**Figure S15.** HRMS (APCI positive mode) of [Cu(tpfc)].	S15
**Figure S16.** HRMS (ESI negative mode) of [Cu-tpfc]$_2$COT.	S16
**Figure S17.** HRMS (ESI negative mode) of [Mn-tpfc]$_2$COT.	S17
**References**	S18

Crystallographic details

The single-crystal of corrole complexes were immersed in paratone–N oil and mounted on a Rigaku XtaLAB Synergy-S at 100 K for Gross 53R and Gross56R and at 140 K for Gross3R.

The single-crystal of corrole complex Gross62 was immersed in paratone–N oil and mounted on a Nonius Kappa CCD at 200 K. Data collection was performed using monochromated Mo Kα radiation λ = 0.71073 Å for the corrole complexes: Gross53R, Gross62, Gross3R and monochromated Cu Kα radiation λ = = 1.54184 Å for the corrole complex Gross56R, using φ and ω scans to cover the Ewald sphere. Accurate cell parameters were obtained with the amount of indicated reflections. Using Olex2,[1] the structure was solved with the olex2.solve[2] structure solution program using Charge Flipping and refined with the ShelXL[3] refinement package using Least Squares minimization. All non-hydrogen atoms were refined with anisotropic displacement parameters. The hydrogen atoms were refined isotropically in calculated positions using a riding model with their U_{iso} values constrained to 1.5 times the U_{eq} of their pivot atoms for terminal sp^3 carbon atoms and 1.2 times for all other carbon atoms. Software used for molecular graphics: Mercury 2022.3.0.

Table S1: Crystallographic information for all complexes.

Crystal data	Gross 53R [Mn^{III}tpfc(py)$_2$]	Gross 62 [Mn^{III}tpfc(py)]$_2$COT	Gross 3R [Mn^{III}tpfc(py)$_2$]$_2$COT	Gross 56R [Cu-tpfc]$_2$COT
CCDC number	2512634	2521077	2512632	2512633
Empirical formula	$C_{56}H_{27}F_{15}MnN_6$	$C_{126}H_{64}F_{30}Mn_2N_{10}$	$C_{94}H_{32}F_{30}Mn_2N_{12}$	$C_{80}H_{26}Cu_2F_{30}N_8$
Formula weight	1123.77	2397.75	2009.19	1796.17
Temperature (K)	100.15	200.15	140.15	100.15
Crystal system	triclinic	triclinic	triclinic	monoclinic
Space group	P-1	P-1	P-1	P21/c
a (Å)	9.70232(16)	11.232(3)	9.5179(4)	21.1899(4)
b (Å)	13.0651(3)	15.573(3)	15.5548(5)	6.76854(11)
c (Å)	19.8836(3)	16.328(3)	18.8011(7)	28.5361(5)
α (°)	74.2633(16)	108.14(4)	74.573(3)	90
β (°)	78.3110(13)	91.30(9)	81.863(3)	102.0144(19)
γ (°)	75.0910(16)	103.08(6)	73.028(3)	90
Volume (Å^3)	2320.32(8)	2630.2(15)	2560.17(17)	4003.14(13)
Z	2	1	1	2
ρ_{calc}(g/cm^3)	1.608	1.514	1.303	1.490
(μ/mm^{-1})	0.394	0.352	0.348	1.722
F (000)	1130.0	1210.0	1000.0	1780.0
Crystal size (mm^3)	0.39 × 0.36 × 0.3	0.33 × 0.3 × 0.15	0.12 × 0.09 × 0.06	0.18 × 0.06 × 0.06
Radiation	MoKα (λ =0.71073)	MoKα (λ =0.71073)	MoKα (λ = 0.71073)	CuKα (λ = 1.54184)
2Θ range (°)	4.3 to 59.918	2.838 to 47.402	4.486 to 60.292	6.86 to 159.48
Wavelength (Å)	0.71073	0.71073	0.71073	1.54184
Reflections collected	33918	7705	32809	20869
R_{int}	0.0270	0.0480	0.0836	0.0444
Data/restraints/parameters	10663/757/679	7705/1293/910	11759/979/713	7574/937/661
Goodness-of-fit on F^2	1.062	1.025	1.034	1.038
R_1, wR_2 [I> 2σ (I)]	0.0556, 0.1633	0.0374, 0.0898	0.0723, 0.1851	0.0656, 0.1736
R_1. wR_2 [all data]	0.0620, 0.1690	0.0447, 0.0936	0.1206, 0.2067	0.0784, 0.1851
Largest diff. peak/hole/e Å^{-3}	1.46/-1.10	0.25/-0.26	0.57/-0.38	1.52/-0.61
Diffractometer	Rigaku XtaLAB Synergy-S	Nonius Kappa CCD	Rigaku XtaLAB Synergy-S	Rigaku XtaLAB Synergy-S

Electrochemical Experiments: The RRDE measurements were performed by a 4-electrode set-up, with 4 mm glassy carbon disc and 7 mm Pt ring as working electrodes. Ag/ AgCl as reference electrode and Pt counter electrode. 10 µL of ink were drop casted on the glassy carbon surface and dried. All ORR experiments were performed in 0.1 M KOH solution. Before each measurement, the solution was purged with nitrogen for 30 minutes and wetting of the working electrode was performed, by 10 CV cycles at 20 mV/s and 15 CV cycles at 100 mV/s, at the potential window of -0.5 to 0 V. Then, the solution was purged with oxygen for 30 minutes. The ring potential was kept at 0.96V vs. RHE. The results are shown at 1000 rpm, 20 mV/s. The collection efficiency, N, of the electrode was calculated by RRDE experiment of aqueous 2 mM $K_3[Fe(CN)_6]$ + 0.1 M KNO_3 under nitrogen atmosphere. It was found to be 0.448.

Electrochemical characterization of the corroles was performed in a solution of 0.5 mM complex in 0.1 M tetrabutylammonium perchlorate (TBAP) in pyridine and acetonitrile, with glassy a carbon working electrode, Ag/ $AgNO_3$ and Pt wire as reference and counter electrodes, respectively. The solution was purged with nitrogen prior to the experiment.

The percentage of peroxide ions is calculated by equation S1:

S1) $\%H_2O_2 = I_R/I_D \times N \times 100\%$

The total number of transferred electrons is calculated by equation S2:

S2) $n = 2 \times I_R/I_D \times N + 4 \times (1 - I_R/I_D \times N)$

where I_R is ring current, I_D is disc current and N is the collection efficiency of the RRDE.[4,5]

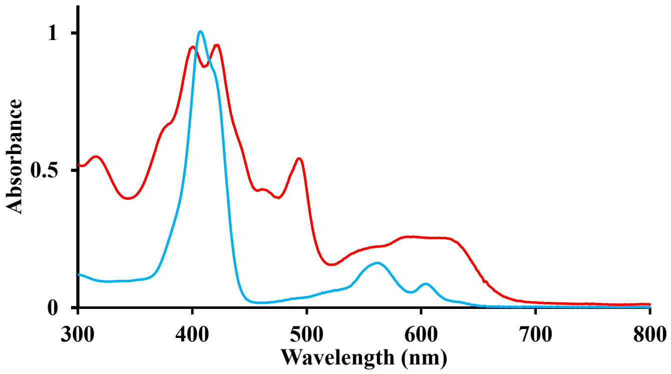

Figure S1: UV-vis (in CH_2Cl_2 at 295 K) spectra of free base H_3tpfc (blue line) and [Mn^{III}tpfc$(py)_2$] (red line).

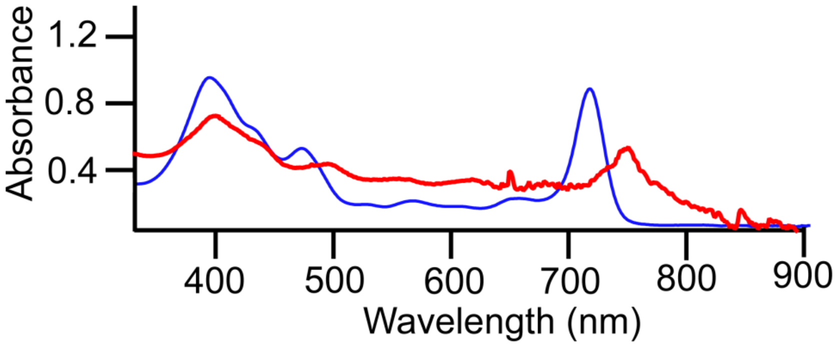

Figure S2. UV-vis (in CH_2Cl_2 at 295 K) spectra of free base dimer (blue line) and [Mn^{III}tpfc(py)$]_2$COT (red line).

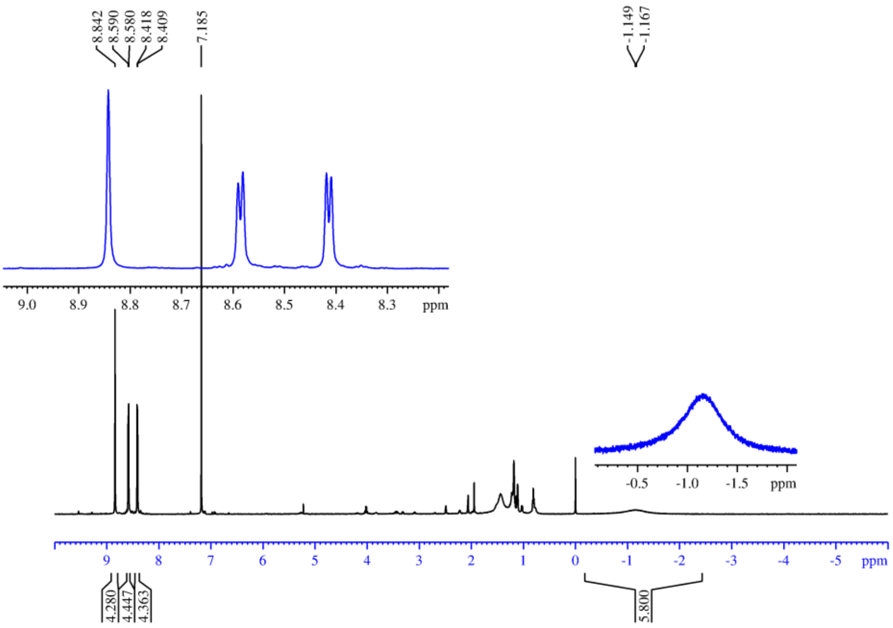

Figure S3. ^{1}H NMR of free-base (H_3tpfc COT) dimer in $CDCl_3$.

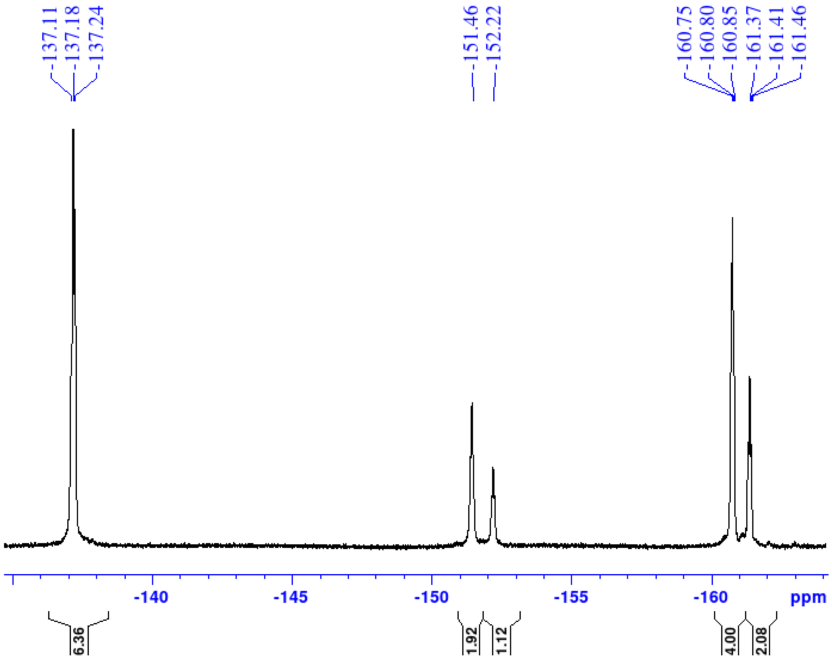

Figure S4. ^{19}F NMR of free-base (H_3tpfc COT) dimer in $CDCl_3$.

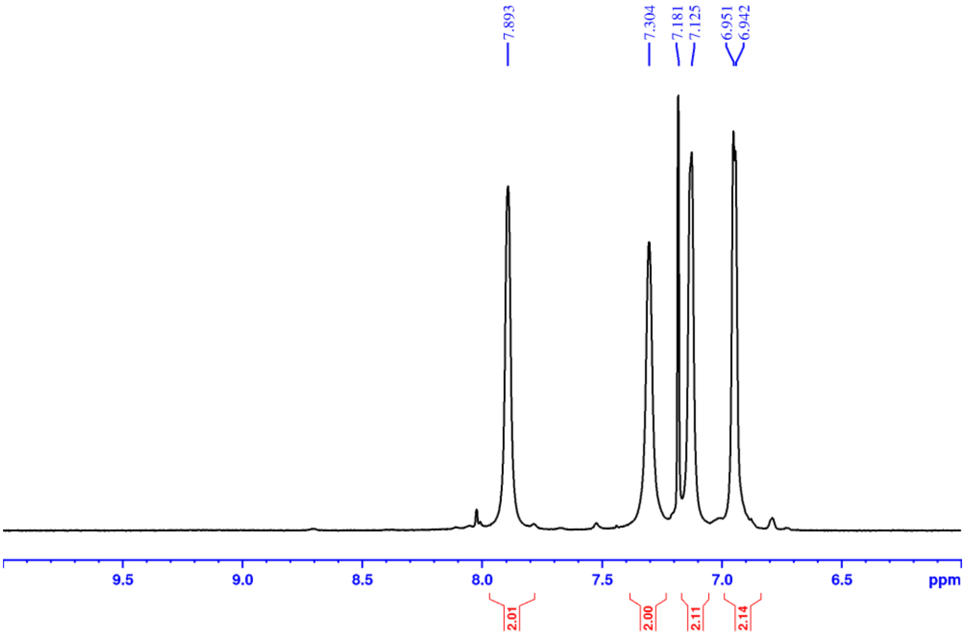

Figure S5. ^{1}H NMR of Cu(tpfc) monomer in $CDCl_3$.

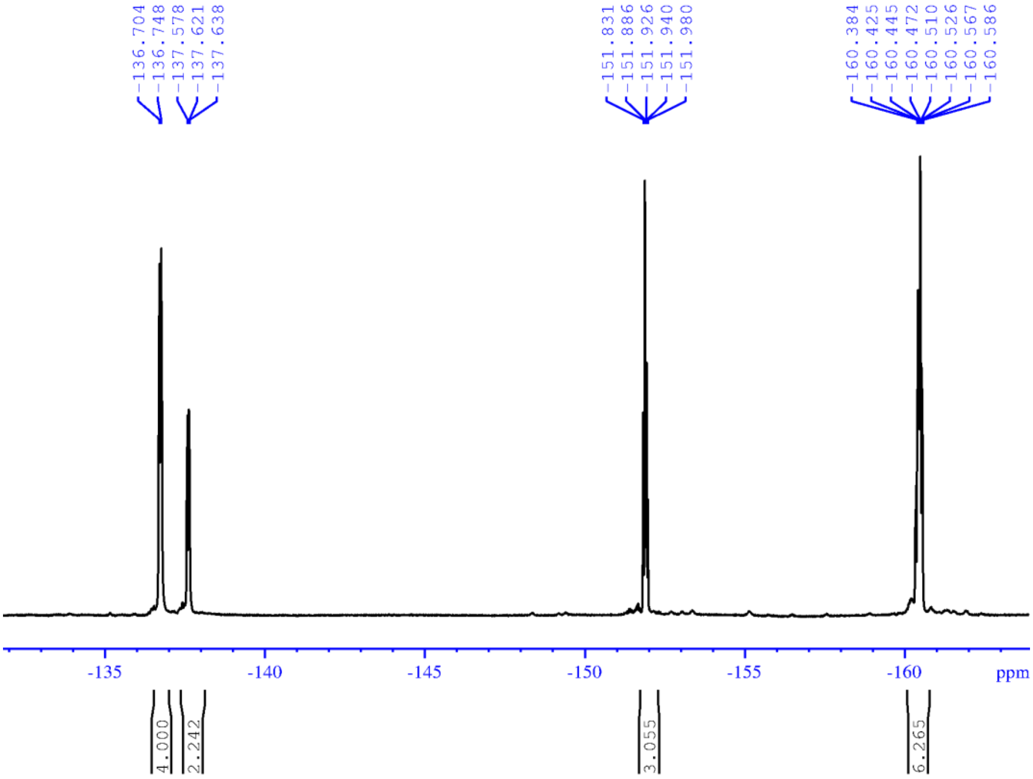

Figure S6. ^{19}F NMR of Cu(tpfc) monomer in $CDCl_3$.

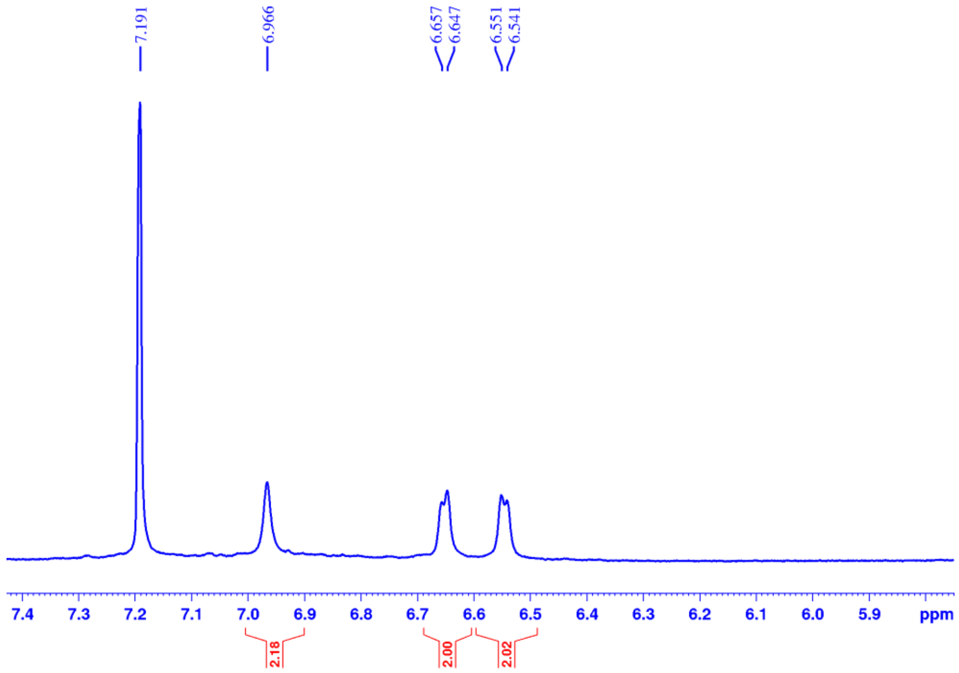

Figure S7. ^{1}H NMR of $[Cu(tpfc)]_2COT$ dimer in $CDCl_3$.

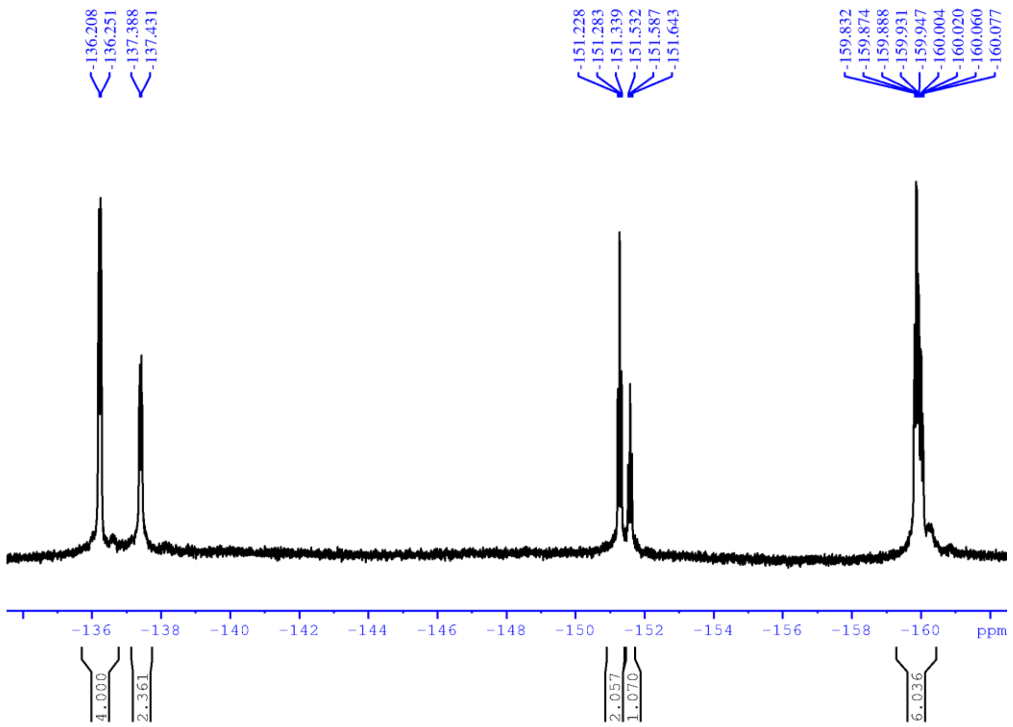

Figure S8. ^{19}F NMR of $[Cu(tpfc)]_2COT$ dimer in $CDCl_3$.

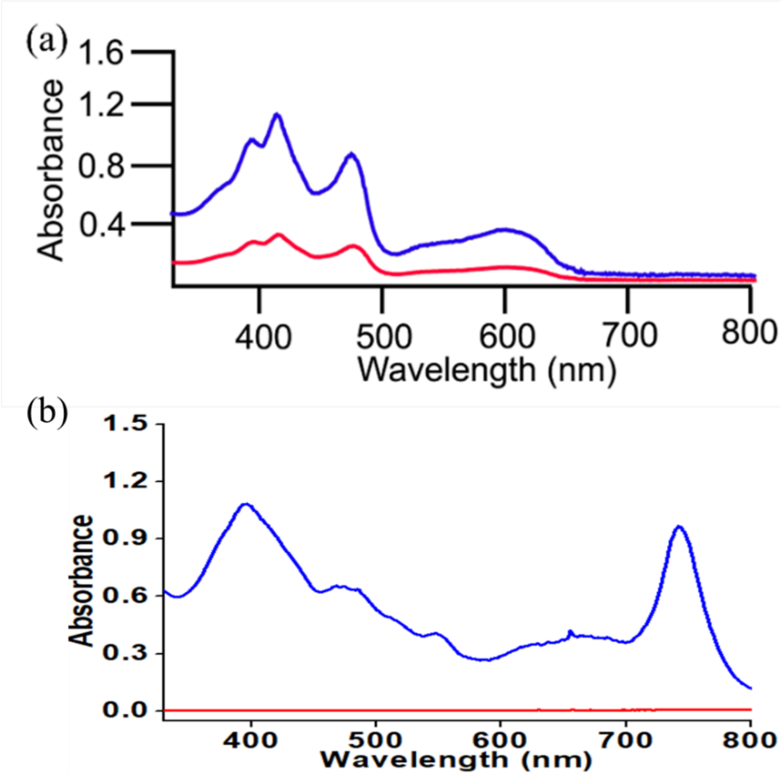

Figure S9. Electronic spectra before (blue traces) and after (red traces) adsorption of 0.8 mg of (a) $[Mn^{III}tpfc(py)_2]$ and (b) $[Mn^{III}tpfc(py)]_2COT$ on 10 mg of BP2000 from 1 mL solution of isopropanol.

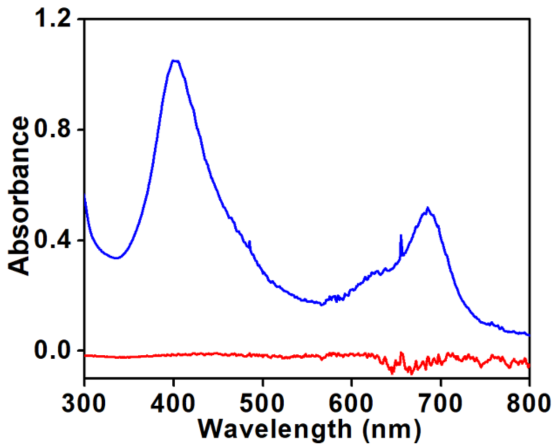

Figure S10. Electronic spectra before (blue traces) and after (red traces) adsorption of 0.8 mg of $[Cu\text{-}tpfc]_2COT$ on 10 mg of BP2000 from 1 mL solution of isopropanol.

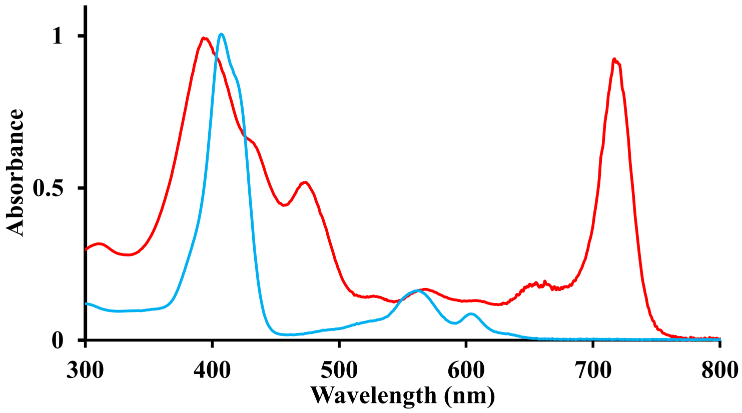

Figure S11. UV-vis (in CH_2Cl_2 at 295 K) spectra of free base H_3tpfc (blue line) and (H_3tpfc COT) dimer (red line).

Display Report

Analysis Info

Analysis Name	D:\Data\Gross\Gr_7776.d	Acquisition Date	15/01/2026 09:17:56
Method	APCI_pos_SolidProbe.m	Operator	Larisa Panz
Sample Name	H3tpfc	Instrument	maXis impact 282001.00128
Comment			

Acquisition Parameter

Source Type	APCI	Ion Polarity	Negative	Set Nebulizer	1.2 Bar
Focus	Active	Set Capillary	4000 V	Set Dry Heater	120 °C
Scan Begin	50 m/z	Set End Plate Offset	-500 V	Set Dry Gas	1.5 l/min
Scan End	2000 m/z	Set Charging Voltage	2000 V	Set Divert Valve	Source
		Set Corona	2000 nA	Set APCI Heater	250 °C

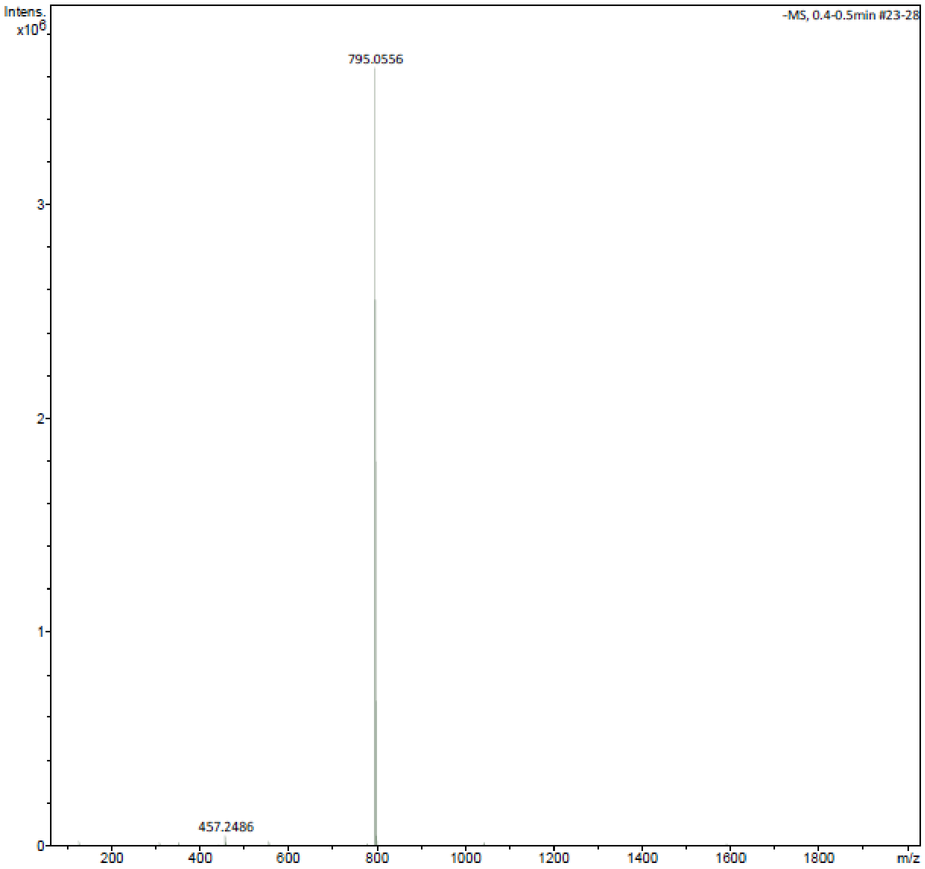

Gr_7776.d

Bruker Compass DataAnalysis 4.2 printed: 15/01/2026 09:21:48 by: Larisa Panz Page 1 of 1

Figure S12. MS (APCI positive mode) of H_3tpfc.

Analysis Info

Analysis Name	D:\Data\Gross\Gr_4813000002.d	Acquisition Date	15/08/2023 11:28:45
Method	APCI_pos_SolidProbe.m	Operator	Larisa Panz
Sample Name	AT-54	Instrument	maXis impact 282001.00128
Comment			

Acquisition Parameter

Source Type	APCI	Ion Polarity	Positive	Set Nebulizer	1.2 Bar
Focus	Active	Set Capillary	3000 V	Set Dry Heater	120 °C
Scan Begin	50 m/z	Set End Plate Offset	-500 V	Set Dry Gas	1.5 l/min
Scan End	2000 m/z	Set Charging Voltage	2000 V	Set Divert Valve	Source
		Set Corona	5000 nA	Set APCI Heater	300 °C

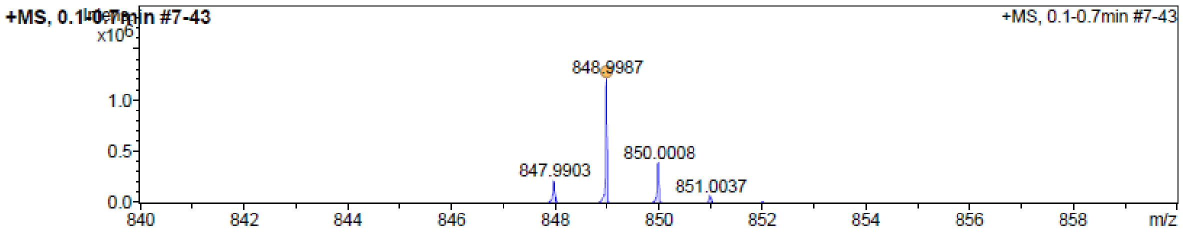

Meas. m/z	#	Ion Formula	m/z	err [ppm]	mSigma	# mSigma	Score	rdb	e⁻ Conf	N-Rule	|err| [mDa]
848.9987	1	C37H9F15MnN4	848.9963	-2.9	47.9	1	100.00	28.0	odd	-	2.4

Figure S13. HRMS (APCI positive mode) of [Mn^{III}tpfc(py)$_2$].

Display Report

Analysis Info

Analysis Name	D:\Data\Gross\Gr_7775.d	Acquisition Date	15/01/2026 09:22:38
Method	APCI_pos_SolidProbe.m	Operator	Larisa Panz
Sample Name	Fused	Instrument	maXis impact 282001.00128
Comment			

Acquisition Parameter

Source Type	APCI	Ion Polarity	Negative	Set Nebulizer	1.2 Bar
Focus	Active	Set Capillary	4000 V	Set Dry Heater	120 °C
Scan Begin	50 m/z	Set End Plate Offset	-500 V	Set Dry Gas	1.5 l/min
Scan End	2000 m/z	Set Charging Voltage	2000 V	Set Divert Valve	Source
		Set Corona	2000 nA	Set APCI Heater	250 °C

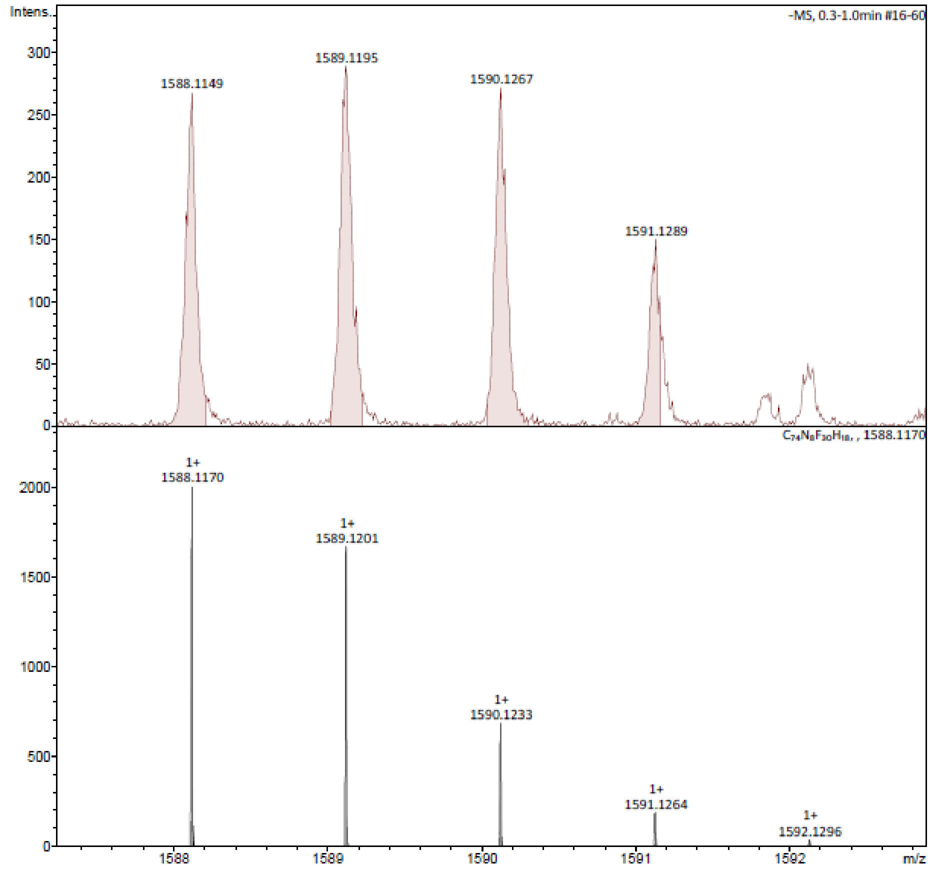

Gr_7775.d

Bruker Compass DataAnalysis 4.2 printed: 15/01/2026 09:27:36 by: Larisa Panz Page 1 of 1

Figure S14. HRMS (APCI positive mode) of (H_3tpfc COT) dimer.

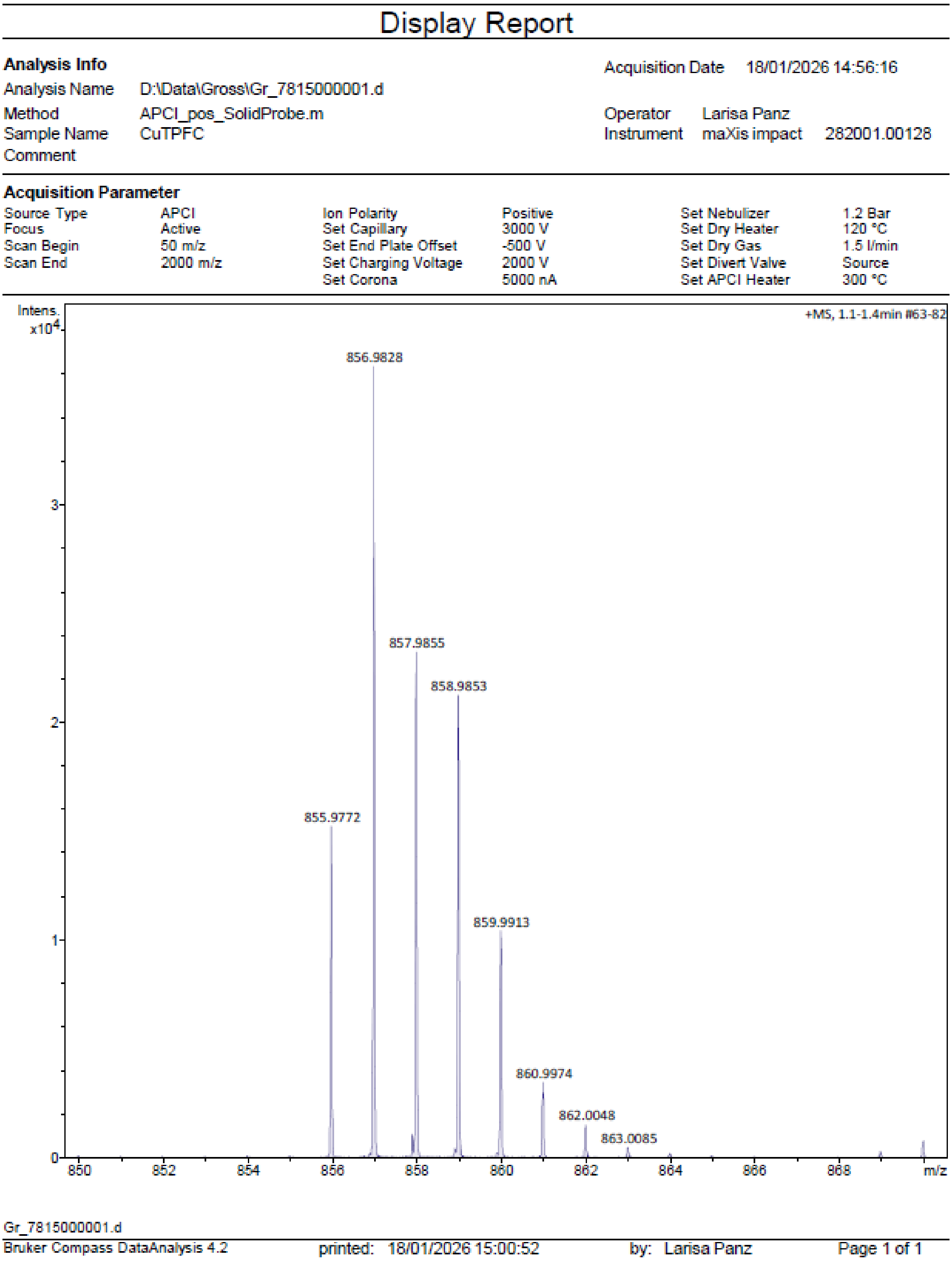

Figure S15. HRMS (APCI positive mode) of [Cu-tpfc].

Display Report

Analysis Info

Analysis Name	D:\Data\Gross\Gr_7803n Cu-Fus_RB6_01_7580.d	Acquisition Date	19/01/2026 14:53:21
Method	direct neg new.m	Operator	Larisa Panz
Sample Name	Gr_7803n Cu-Fus	Instrument	maXis impact 282001.00128
Comment			

Acquisition Parameter

Source Type	ESI	Ion Polarity	Negative	Set Nebulizer	3.0 Bar
Focus	Active	Set Capillary	3900 V	Set Dry Heater	180 °C
Scan Begin	50 m/z	Set End Plate Offset	-500 V	Set Dry Gas	8.0 l/min
Scan End	3500 m/z	Set Charging Voltage	2000 V	Set Divert Valve	Source
		Set Corona	0 nA	Set APCI Heater	0 °C

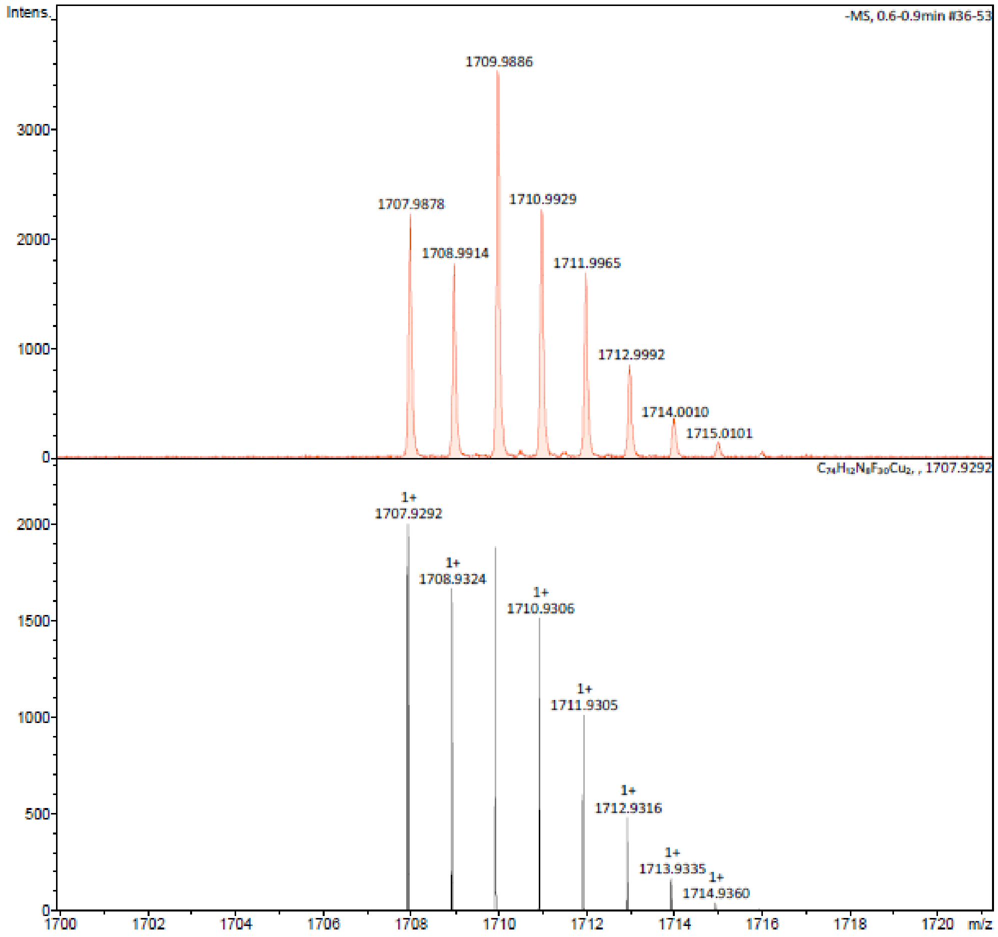

Gr_7803n Cu-Fus_RB6_01_7580.d

Bruker Compass DataAnalysis 4.2 printed: 20/01/2026 13:08:28 by: Larisa Panz Page 1 of 1

Figure S16. HRMS (ESI negative mode) of $[Cu\text{-}tpfc]_2COT$.

Display Report

Analysis Info

Analysis Name	D:\Data\Gross\Gr_7802n Mn-Fus_RB5_01_7579.d	Acquisition Date	19/01/2026 14:48:51
Method	direct neg new.m	Operator	Larisa Panz
Sample Name	Gr_7802n Mn-Fus	Instrument	maXis impact 282001.00128
Comment			

Acquisition Parameter

Source Type	ESI	Ion Polarity	Negative	Set Nebulizer	3.0 Bar
Focus	Active	Set Capillary	3900 V	Set Dry Heater	180 °C
Scan Begin	50 m/z	Set End Plate Offset	-500 V	Set Dry Gas	8.0 l/min
Scan End	3500 m/z	Set Charging Voltage	2000 V	Set Divert Valve	Source
		Set Corona	0 nA	Set APCI Heater	0 °C

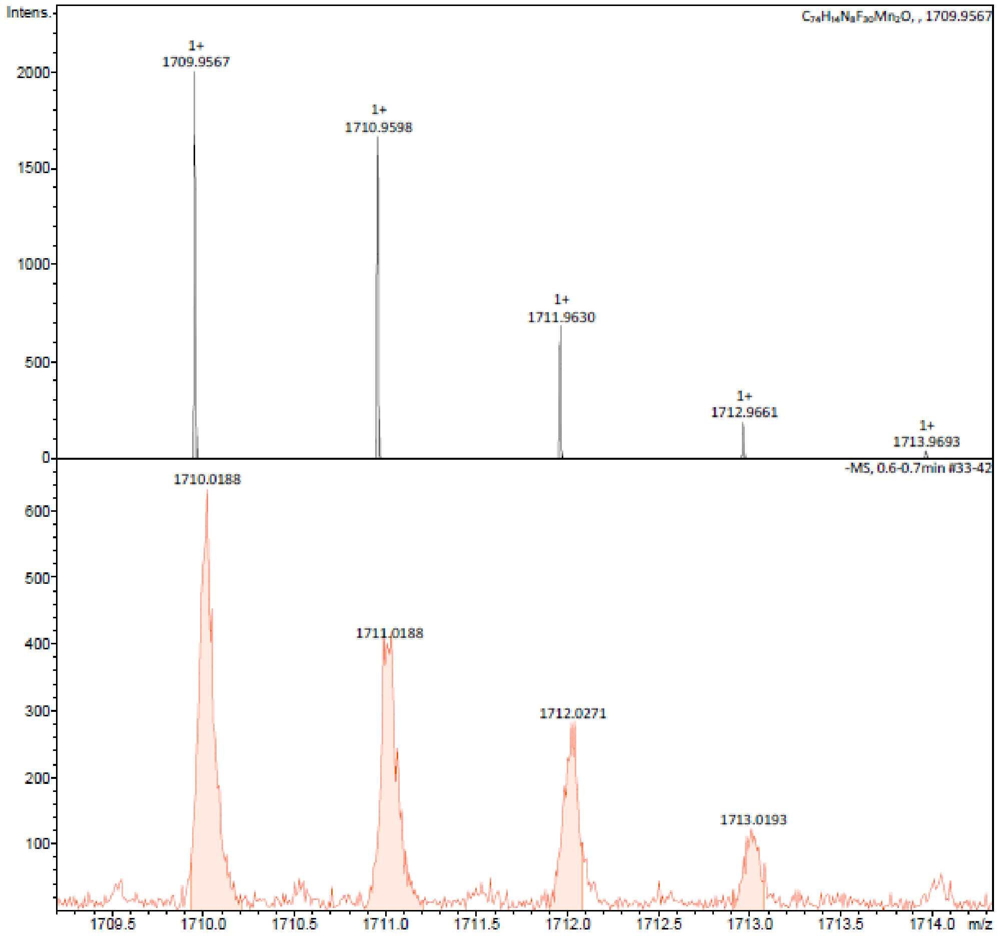

Gr_7802n Mn-Fus_RB5_01_7579.d

Bruker Compass DataAnalysis 4.2 printed: 21/01/2026 08:33:43 by: Larisa Panz Page 1 of 1

Figure S17. HRMS (ESI negative mode) of $[Mn\text{-}tpfc]_2COT$.

References:

(1) Dolomanov, O. V; Bourhis, L. J.; Gildea, R. J.; Howard, J. A. K.; Puschmann, H. OLEX2: A Complete Structure Solution, Refinement and Analysis Program. *J. Appl. Crystallogr.* **2009**, *42*, 339–341.

(2) Bourhis, L. J.; Dolomanov, O. V; Gildea, R. J.; Howard, J. A. K.; Puschmann, H. The Anatomy of a Comprehensive Constrained, Restrained Refinement Program for the Modern Computing Environment–Olex2 Dissected. *Acta Crystallogr. Sect. A Found. Adv.* **2015**, *71*, 59–75.

(3) Sheldrick, G. M. Crystal Structure Refinement with SHELXL. *Acta Crystallogr. Sect. C Struct. Chem.* **2015**, *71*, 3–8.

(4) Liberman, I.; Shimoni, R.; Ifraemov, R.; Rozenberg, I.; Singh, C.; Hod, I. Active-Site Modulation in an Fe-Porphyrin-Based Metal–Organic Framework through Ligand Axial Coordination: Accelerating Electrocatalysis and Charge-Transport Kinetics. *J. Am. Chem. Soc.* **2020**, *142*, 1933–1940.

(5) Honig, H. C.; Friedman, A.; Zion, N.; Elbaz, L. Enhancement of the Oxygen Reduction Reaction Electrocatalytic Activity of Metallo-Corroles Using Contracted Cobalt(III) CF_3-Corrole Incorporated in a High Surface Area Carbon Support. *Chem. Commun.* **2020**, *56*, 8627–8630.
